# Remineralization of molar incisor hypomineralization (MIH) with a hydroxyapatite toothpaste: an in-situ study

**DOI:** 10.1038/s41405-022-00126-4

**Published:** 2022-12-10

**Authors:** Bennett Tochukwu Amaechi, Rayane Farah, Jungyi Alexis Liu, Thais Santiago Phillips, Betty Isabel Perozo, Yuko Kataoka, Frederic Meyer, Joachim Enax

**Affiliations:** 1 Department of Comprehensive Dentistry, School of Dentistry, University of Texas Health San Antonio, 7703 Floyd Curl Drive, San Antonio, TX 78229-3900 USA; 2 Department of Developmental Dentistry, School of Dentistry, University of Texas Health San Antonio, 7703 Floyd Curl Drive, San Antonio, TX 78229-3900 USA; 3 Dr. Kurt Wolff GmbH & Co. KG, Research Department, Johanneswerkstr. 34-36, 33611 Bielefeld, Germany

**Keywords:** Paediatric dentistry, Minimal intervention dentistry

## Abstract

**Aim:**

This randomized, double-blind, crossover, in-situ study, compared the efficacy of toothpastes based on microcrystalline hydroxyapatite (HAP; fluoride-free) or fluoride, in remineralizing molar incisor hypomineralization (MIH).

**Methods:**

Two lesion-bearing enamel blocks were produced from each of thirty extracted permanent molars diagnosed with MIH. Sixty produced blocks were randomly assigned to two groups (30/group): 20% HAP or 1450 ppm fluoride toothpaste. Each group was subdivided into, etched (*n* = 20), with lesion surface treated with 32% phosphoric acid-etchant for 5 s, and unetched (*n* = 10). Blocks were cemented into intra-oral appliances (2 blocks/appliance) worn full-time by 15 subjects. Subjects used the toothpastes in a two-phase crossover manner, lasting 14 days per phase, after one-week washout period. Baseline and post-treatment mineral density (MD) was quantified using microcomputed tomography.

**Results:**

Overall, both groups showed statistically significant (paired *t*-test; *p* < 0.001) net-gain when MD was compared pre-treatment and post-treatment. HAP: pre-treatment (1.716 ± 0.315) and post-treatment (1.901 ± 0.354), Fluoride: pre-treatment (1.962 ± 0.363) and post-treatment (2.072 ± 0.353). Independent *t*-test demonstrated a practically significantly (≥10%) higher percentage remineralization with HAP toothpaste (26.02 ± 20.68) compared with fluoride toothpaste (14.64 ± 9.60). Higher percentage remineralization was observed in etched than unetched samples.

**Conclusion:**

The tested toothpaste based on hydroxyapatite can remineralize MIH lesions. Pre-treating the tooth surface with acid-etchant enhanced remineralization.

## Introduction

Molar incisor hypomineralization (MIH) is a qualitative enamel defect affecting at least one first permanent molar, with a frequently associated affection of the permanent maxillary incisors or less likely the permanent mandibular incisors [1]. Clinically, MIH is diagnosed when a first permanent molar presents with a demarcated opacity, larger than 1 mm, that can vary in color from white to yellow and even brown [2, 3]. Additionally, post-eruptive enamel breakdown, atypically sized, shaped, and extended restorations, or a history of extraction of first permanent molars in an otherwise sound dentition, are also diagnostic criteria of MIH [2]. The location, asymmetry and varying severity of the lesions, makes it possible to distinguish them from other diffuse enamel mineralization defects such as fluorosis [4]. The severity of the affection can be divided into 2 categories, mild and moderate/severe. These categories are distinguishable clinically based on the presence or absence of enamel breakdown, the degree of hypersensitivity and the esthetic concerns [3]. This classification is also correlated with reduced levels of mineral density of the affected enamel [5]. In fact, the mineral density of sound enamel ranges from 2.4 to 2.8 g/cm^3^ [5, 6]. In contrast, mild hypomineralization lesions were found to have a mineral density of approximately 2.24 g/cm^3^, while moderate to severe lesions have mean mineral density values between 1.67 and 1.93 g/cm^3^ [5].

Recent global studies reported a prevalence of MIH of 13.1% and 14.2% worldwide [7, 8]. The variability of the findings is due to the difference in the index used, the age of the subjects and the region studied. MIH is multifactorial disorder resulting from genetic as well as systemic environmental factors. Perinatal and postnatal factors appear to be more strongly associated with the appearance of MIH than prenatal factors [9]. In fact, premature birth, type of delivery and associated complications increase the risk of developing MIH [9]. Similarly, childhood illnesses such as ear or respiratory infections, antibiotic use, hypoxia, gastric disorders, dioxin exposure are also implicated in increased incidence of MIH [3, 9]. If these conditions develop in the early childhood years, when the crowns of the permanent molars and incisors are undergoing mineralization, they predispose the child to the occurrence of MIH [3, 10]. Consequently, the time of onset, span and severity of the conditions dictate the pattern and intensity of the lesions.

Management of patients with MIH is complicated by the penetration of bacteria through the often-exposed dentinal tubules at the level of the hypomineralized lesion [11]. This bacterial infiltration is responsible for subclinical inflammation of the pulp and hypersensitivity that lead to negligence of oral hygiene by the child and a higher anesthetic threshold [11, 12]. As for treatment needs, studies have shown that patients with MIH lesions are ten times more likely to need dental treatment due to a higher risk of developing caries [12]. Additionally, esthetic and functional concerns are frequent in these patients and are associated with a lower oral health related quality of life (OHRQoL) [13]. The treatment of teeth with hypomineralization defects is two-fold, i.e., preventive and interventional. Therapeutic interventions must take place when the defects are considered severe and when the teeth display enamel breakdown, decay or signs of pulpal inflammation/infection [14]. The therapeutic approach includes direct restoration with glass ionomer, composite resin or preformed metallic crowns for posterior teeth and micro-abrasion with resin infiltration or composite restoration for anterior teeth [14]. In more advanced cases, when the affected teeth are deemed non-restorable, extraction with space management is indicated [3]. However, when MIH diagnosis is established early, the prevention of the progression of the defect is recommended by using fissure sealants, desensitizers and remineralizing agents [14–16]. In fact, increasing the mineral content of MIH-affected enamel by applying remineralizing agents to reach a threshold close to healthy teeth can improve its physical properties, hence improving the esthetic and functional outcomes.

Several techniques pertaining to remineralization of MIH lesions have been described in the literature including, the application of toothpastes and varnishes containing fluoride (with or without tricalcium phosphate), casein phosphopeptide-amorphous calcium phosphate (CPP-ACP) or casein phosphopeptide-amorphous calcium fluoride phosphate (CPP-ACFP) [17–20].

Toothpastes based on hydroxyapatite (HAP) were shown to have comparable effects to fluoride toothpastes in remineralization of initial caries lesions [21–26] and prevention of dental decay while excluding the risk of fluorosis [27] and other fluoride-associated side effects [28–30]. The synthesized particles of HAP are structurally similar to the crystallites found in human enamel [31], have analogous properties, and can readily penetrate enamel surface [32]. Besides caries prevention [22, 23, 30, 33] and remineralization, HAP offers further benefits like reducing dentin hypersensitivity [34, 35], biofilm management [36, 37], erosion protection [38, 39], and whitening [40, 41]. In contrast to many other ingredients in oral care, HAP can act as a source of calcium and phosphate ions as well as an acid buffer in bacterial biofilms [42, 43]. The first randomized clinical trial using a fluoride-free HAP toothpaste in the field of MIH was performed in Germany [16]. It was shown that a HAP-toothpaste reduced MIH-associated hypersensitivity in children. Compared to the fluoride control group, children using the HAP-toothpaste had the tendency to be less affected by MIH-associated hypersensitivity [16]. HAP shows a high biocompatibility and can be easily applied as toothpastes and mouthwashes during daily oral hygiene [16, 44–46].

The remineralization efficacy of HAP has been clearly shown on enamel and dentin in various studies [22, 47, 48], but to date, the use of HAP as remineralization agent for MIH defects has not been investigated. For this reason, the objective of the present in-situ study was to determine the efficacy of a fluoride-free toothpaste based on 20% HAP (Bioniq^®^ Repair-Zahncreme; Dr. Kurt Wolff GmbH and Co. KG, Bielefeld, Germany) in promoting remineralization of teeth with MIH defects, comparing it with a fluoride toothpaste containing 1450 ppm fluoride as sodium fluoride (Colgate Komplett 8 Zahnpasta; Colgate-Palmolive Company, Hamburg, Germany). It is pertinent to mention that in this in situ-study, we applied the concept of practical significance, which is concerned with whether the result is useful in the real world, and it is adapted to deal with cases where groups have different standard deviations [49, 50]. A practical significance was established if the difference in the mean percentage remineralization between the two toothpaste formulations is ≥10% with a Cohen’s *d* large effect size. Our null hypotheses were that (1) none of the two toothpaste formulations would achieve mean percentage remineralization of hypomineralized enamel that is greater than zero, and (2) there would not be a practical significant difference between the efficacy of the HAP toothpaste and the fluoride toothpaste in remineralizing MIH lesions.

## Materials and methods

### Production of samples

Following consent from the donors, freshly extracted permanent teeth were collected from the dental clinics of the University of Texas Health San Antonio (UTHSA). The teeth were cleaned of debris/stains and examined with a transilluminator (Microlux™ Transilluminator, AdDent Inc., Danbury, CT, USA) by 2 trained examiners (RF & BP). Teeth with MIH but free of caries or other malformations were selected and cleaned with pumice to remove the pellicle. MIH lesions were confirmed by experienced pediatric dentist (JAL). The diagnosis of the MIH lesion was established if the opacity presented as a well-defined alteration of the translucency of the enamel, was larger than 1 mm, had a color varying from creamy white to yellow to brown and was located on smooth buccal or lingual surfaces of a permanent first molar [2, 3]. These criteria differentiate MIH from other hypomineralization lesions such as enamel hypoplasia which present ill-defined margins, traumatic hypomineralization that occurs on anterior teeth, or white spot lesions that are found on plaque retentive surfaces or around orthodontic brackets [4, 10]. Using a water-cooled diamond wire saw, an MIH-bearing tooth block, measuring approximately 2 mm length × 2 mm width × 1.5 mm thickness, was produced from each hypomineralized lesion.

A total of 60 blocks were produced and randomly assigned to two categories: etched and unetched, in a ratio of 2:1 resulting in 40 etched blocks and 20 unetched blocks. The enamel surface of each block in etched category was treated with 32% phosphoric acid-etchant (Uni-Tech, BISCO, Inc., USA) for 5 s followed by rinsing with an air/water syringe for 20 s. The unetched group did not undergo any surface treatment.

### Measurement of baseline mineral density of the tooth blocks

Micro-computed X-ray tomography (µ-CT) is a high resolution, non-destructive radiographic method of analysis that generates qualitative images and quantitative analysis of the mineral density (MD) in tooth samples [5, 51]. The scanning protocols were performed as described earlier [6] in a desktop scanner (Bruker SkyScan 1172, Kontich, Belgium) with a 0.5 mm aluminum filter at 60 kV, 167 mA beam intensity and a 0.35° rotation step with 1090 ms exposure time at each step. The image pixel size was 6 microns. Samples were stabilized inside the tube using spongy foams to prevent movement during scanning and allow their evaluation over time in the same position. 150 microns (0.15 mm) diameter volumes of interest (VOI), the borders of which extends from along the surface of the enamel down to a depth of 150 microns into the enamel towards the dentin, was determined. Enamel mineral density (MD) were measured in this VOI. A series of hydroxyapatite (HA) phantoms (Himed, Bethpage, NY, USA) with varying densities were also scanned under the same conditions and settings as the test blocks and were used to calibrate the enamel density measurements. The samples were reconstructed using NRecon (Bruker SkyScan, Aartselaar, Belgium) using a polynomial correction algorithm [6]. Using the density measuring program, Bruker-MicroCT CT-Analyser software, the linear attenuation coefficient (LAC, cm^−1^) was measured from the tomographic images of each sample and was used to determine the MD [51].

### Recruitment of study subjects

This was a double-blind, randomized, crossover, single center, controlled in-situ study. The primary outcome to be examined was the percentage remineralization (i.e., percentage gain in MD) of the MIH lesions measured relative to the baseline MD of the MIH lesions. The approval (#: 20210570HU) of the UTHSA Institutional Review Board was obtained. Subjects were recruited from among the patients attending the dental clinics of the UTHSA School of Dentistry from the local San Antonio area. This in situ study was conducted in accordance with the ethical standards outlined in the 1964 Declaration of Helsinki and its later amendments, and in compliance with International Conference on Harmonization (ICH) Good Clinical Practice Guidelines. At the screening visit, each subject completed a medical/dental history and read and signed an informed consent form. Following consent, a visual oral health exam was performed by a licensed dentist (RF).

**Fig. 1:**
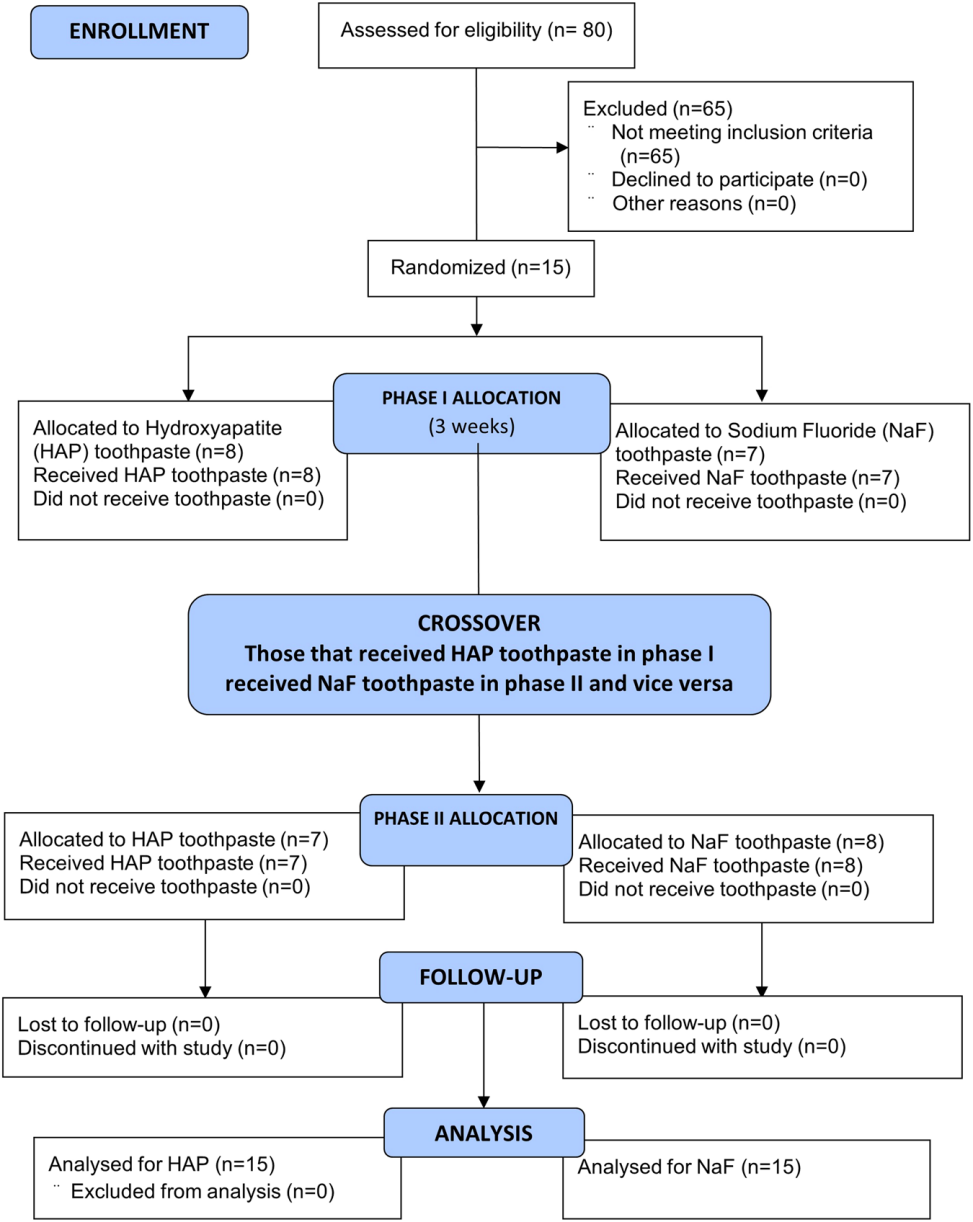
Flow diagram showing the stepwise methodology. The 15 participants who completed the study, received the two interventions in a crossover design as phase I and II, each phase started with a 1-week washout period and was followed by 2-weeks of study toothpaste use.

Fifteen subjects were recruited into the study (Fig. 1). The inclusion criteria were being between 18 to 60 years old, in good general health, and with no known history of allergy to personal care/consumer products (Table 1). Additionally, participants had to have a minimum of 20 natural uncrowned teeth (excluding third molars), no active unrestored cavities, and a normal salivary flow rate (stimulated and unstimulated flow of ≥0.7 mL/min and ≥0.2 mL/min, respectively) ascertained from a preliminary sialometry test [52]. They also had to give written informed consent, be available throughout entire study, and be willing to wear intra-oral appliance 24 h per day and use only assigned products for oral hygiene throughout the duration of the study.

**Table 1:** List of inclusion and exclusion criteria.

Inclusion criteria	Exclusion criteria
18 to 60 years old, in good general health	Having a medical condition that requires premedication prior to dental visits/procedures
No known history of allergy to personal care/consumer products	Having history of allergy to common toothpaste ingredients
A minimum of 20 natural uncrowned teeth (excluding third molars)	Not enough teeth to secure the intra-oral appliance
No active unrestored cavities	Wearing orthodontic appliances
A normal salivary flow rate (stimulated and unstimulated flow of ≥ 0.7 mL/min and ≥ 0.2 mL/min, respectively)	Having an impaired salivary function or Using drugs that can affect salivary flow
Willing to give written informed consent	Having diseases of the soft or hard oral tissues
Willing to be available throughout entire study	Using antibiotics one month prior to or during this study,
Be willing to wear intra-oral appliance 24 h per day	Participating in another clinical study one week prior to the start of the washout period or during this study period
Willing to use only assigned products for oral hygiene throughout the duration of the study	Using tobacco products
	Having an advanced periodontal disease
	Having a compromised immune system (HIV, AIDS, immuno-suppressive drug therapy).

Potential subjects were excluded if they have advanced periodontal disease, a medical condition that requires premedication prior to dental visits/procedures, an impaired salivary function, orthodontic appliances, not enough teeth to secure the intra-oral appliance, or diseases of the soft or hard oral tissues (Table 1). Other excluding criteria were using drugs that can affect salivary flow, using antibiotics one month prior to or during this study, participating in another clinical study one week prior to the start of the washout period or during this study period, using tobacco products, having history of allergy to common toothpaste ingredients, and having a compromised immune system (HIV, AIDS, immuno-suppressive drug therapy) as determined by review of medical history.

### Construction of the intra-oral appliance

Following recruitment, an impression of each subject’s lower dentition was taken using an alginate impression material. A dental technician fabricated a lower removable intra-oral appliance. All tooth blocks were then covered with polyester gauze (Bard Peripheral Vascular, Inc., USA), which facilitated plaque retention on the surface of the tooth blocks on intra-oral exposure [21]. Each of the MIH-lesion-bearing blocks were mounted on each side within the acrylic portion of the removable appliance, using Intermediate Restorative Material (IRM) cement (fluoride–free). All appliances were sterilized with ethylene oxide prior to delivery to the subject.

### Test product description and labeling

The toothpastes used for treatments (Table 2) were a 20% microcrystalline HAP toothpaste (test), a 1450 ppm fluoride (provided as NaF) toothpaste (comparator), and a non-fluoride, non-HAP toothpaste (washout). The test and comparator products were coded by the manufacturing/packaging company that retained the code until the completion of the study and data interpretation. The experiment consisted of two distinct treatment phases, Phase 1 and Phase 2, during which subjects were exposed to one of the two products in a randomized crossover design.

**Table 2:** The composition of the study toothpastes.

Toothpaste	*Composition*
Bioniq® Repair-Zahncreme (20% hydroxyapatite; fluoride-free)	*Aqua, Hydroxyapatite, Hydrated Silica, Glycerin, Sorbitol, Aroma, Sodium Myristoyl Sarcosinate, Cellulose Gum, Silica, Tetrapotassium Pyrophosphate, Zinc PCA, Sodium Methyl Cocoyl Taurate, Sodium Saccharin, Citric Acid, Phenoxyethanol, Propylparaben, Methylparaben, Sodium Benzoate, Benzyl Alcohol, Limonene*.
Colgate Komplett 8 Zahnpasta (1450 ppm F^-^ as sodium fluoride)	*Aqua, Sodium Fluoride, Sorbitol, Hydrated Silica, Glycerin, Sodium Lauryl Sulfate, PEG-12, Aroma, Celluluse Gum, Sodium Saccharin, CI 74160, CI 74260, CI 77891*.

### Study grouping

The 40 etched blocks were randomly assigned to two product groups: HAP or fluoride toothpastes (20 blocks/group). The blocks were assigned such that the mean MD of the two groups did not differ significantly. Similarly, the etched blocks were randomly assigned to the same HAP or fluoride groups (10 blocks/group), and again, the assignment was such that the mean MD of the two unetched groups did not differ significantly. Thus, each product group has 30 MIH-bearing blocks (20 etched and 10 unetched). The combined mean MD of the etched and unetched blocks in HAP group (*n* = 30) did not differ from that of the fluoride group (*n* = 30). Following the grouping, enrolled subjects were identified with code numbers (CP01 to CP15) and then randomly assigned to one of two treatment sequences: use of HAP toothpaste in phase 1 then NaF toothpaste in phase 2 or vice versa.

### Study procedure and patient instructions

Prior to each 2-week treatment phase, subjects completed a 1-week washout period. This period allows for attenuation of any residual effect of the subject’s previously used toothpaste. During this period, no appliance was worn, and subjects used only the provided washout toothpaste and a soft-bristled toothbrush twice daily. Following the washout period, the intra-oral appliance was fitted to each subject by a qualified dentist (RF). However, before fitting the appliance, every subject received professional teeth cleaning to ensure that every subject started at the same baseline oral hygiene. Immediately after fitting of the first appliance (on day 1 of the first treatment phase), each subject received a soft bristled manual toothbrush for use throughout the duration of the study and a toothpaste according to the treatment phase. They made the first use of the test product under the supervision of the study coordinator. For the remainder of the study, subjects completed the procedure at home and as instructed.

The subjects were instructed to brush their teeth with the appliance in the mouth, two times daily, for 3 min on each brushing episode, in the morning after breakfast and last thing before bed, followed by rinsing with 10 mL of water. In dispensing the toothpaste onto the toothbrush, the subjects had to fill the toothbrush surface from end to end but no more than one ribbon of toothpaste. The subjects were advised not to brush directly on the blocks but rather to brush around the blocks to prevent disruption of the plaque. Subjects were required to neither eat nor take any drink for at least 30 min after brushing and were also instructed to remove the appliance while eating. A timer and measuring cup were provided to each subject. To monitor product usage and compliance, a diary was provided to each subject and checked at every visit, to record the number of tooth-brushings performed each day and the time it was done. Further, subjects were instructed to return the remaining toothpaste after each washout or treatment phase. The weight of toothpaste was measured before and after each treatment phase. Over the study period, all subjects had to maintain their normal dietary habits.

On day 15, the subject, without using the product that morning, arrived at the clinic, and the tooth block was harvested and sent to the laboratory for µCT analysis. Then the subject was given washout toothpaste and a soft-bristle toothbrush to undergo another 7-day washout period without an appliance. After completion of the second washout period, subjects returned to the clinic, and the appliance, with new MIH-bearing tooth blocks mounted, was fitted to the subject for the phase 2 treatment period. This procedure was then repeated until the 2-week treatment phase was completed, and each subject had gone through the two arms of the study.

Upon completion of the treatment phases, the post-remineralization mineral density (MDt) of all the MIH-lesion-bearing blocks exposed intra-orally for remineralization was assessed with µCT. This process generated the pre-test (MDb) and post-test (MDt) mineral density of the lesions and their associated images.

At every visit, the dental examiner visually examined the soft and hard tissues of the oral cavity and peri-oral area using a dental light and dental mirror. Additionally, subjects were asked about and examined for any adverse events. Subjects were able to call and request a visit for any concern of potential adverse event.

### Data handling

Using the µ-CT images, the pattern and the extent of remineralization produced within each lesion by each treatment product was examined and described. This was clearly shown by comparing the pre- and post-test images side-by-side. For all calculations, the absolute MD was measured and used. The mean values of the MDb and MDt for each product group was compared using paired t-test to determine any significant change (remineralization) made by the test product (intra-group comparison). However, to make comparisons between the two products (intergroup comparison), percentage change in MD calculated relative to the baseline value was determined for each product group as follows.

% Change in MD (%ΔMD) = [(MDt – MDb)/MDb] x 100.

### Sample size calculation

The power analysis and sample size calculation were performed using nQuery Advisor software (Statistical Solutions, Cork, Ireland). The sample size for this study was based on the primary efficacy outcome, %ΔMD, following 2 weeks of treatment. A sufficient number of subjects were screened to randomize 15 subjects with the intention that approximately 15 subjects complete all study treatments and be evaluable for the efficacy analysis. With these conditions met, the study has 80% power at the 5% significance level, using two-sided testing, to detect a mean treatment difference in %ΔMD of approximately 8.1% assuming a within subject standard deviation of approximately 12.6%. In the absence of available 2-week data from previous mineral density studies, the within subject standard deviation has been estimated using 14-day post-treatment data from previous results obtained by this research group in a demineralization and remineralization study using HAP toothpaste [21]. In that study the mean % remineralization was equal to 30.3 with a standard deviation equal to 16.3.

### Statistical analysis

All analyses were performed using SPSS 28. The data was analyzed at three levels: all data combined, etched data only, and unetched data only. Paired samples *t*-test, comparing the mean values of the pre-treatment and the post-treatment MD for each product (Intra-group comparison), was used to address the first null hypothesis that none of the two toothpaste formulations would promote remineralization of hypomineralized enamel that is significantly greater than zero. The statistical significance for each analysis was established at an alpha level of α = 0.05. The mean percentage remineralization was determined for each product group as a percentage of pre-treatment MD. Using their mean percentage remineralization, the two product groups were compared (inter-group comparison) by independent group *t*-test, to address the second hypothesis that there would not be a practical significant difference between the efficacy of the two products in remineralizing MIH lesions. As stated earlier in the ‘Introduction’ section, in this comparison we applied the concept of practical significance, which adapted to deal with cases where groups have different standard deviations. In the present study, a practical significance was established if the difference in the mean percentage remineralization between the two toothpaste formulations is ≥10% with a Cohen’s *d* large effect size.

Preliminary analyses were conducted to explore the dataset and to check for assumptions violations. Specific t-test assumptions tests including tests for independence, normality and extreme outliers were carried out. The assumptions of independence and that of normality were met. The normality assumption was tested using the histogram, Q-Q plot and the Shapiro-Wilk’s test from the tests of normality table and all confirmed that the normality assumption was met for each variable at the alpha level of α = 0.05.

## Results

Fifteen subjects with a mean (SD) age of 50.2 (11.16) years were recruited and all of them completed both arms of the study. The ethnic distribution of the subjects was as follows: Hispanic 10 (66.6%), Black (not Hispanic) 1 (6.7%), White (not Hispanic) 3 (20%), and others 1 (6.7%). There were no incidents of adverse effects reported by the subjects or observed clinically during the period of the study.

**Fig. 2:**
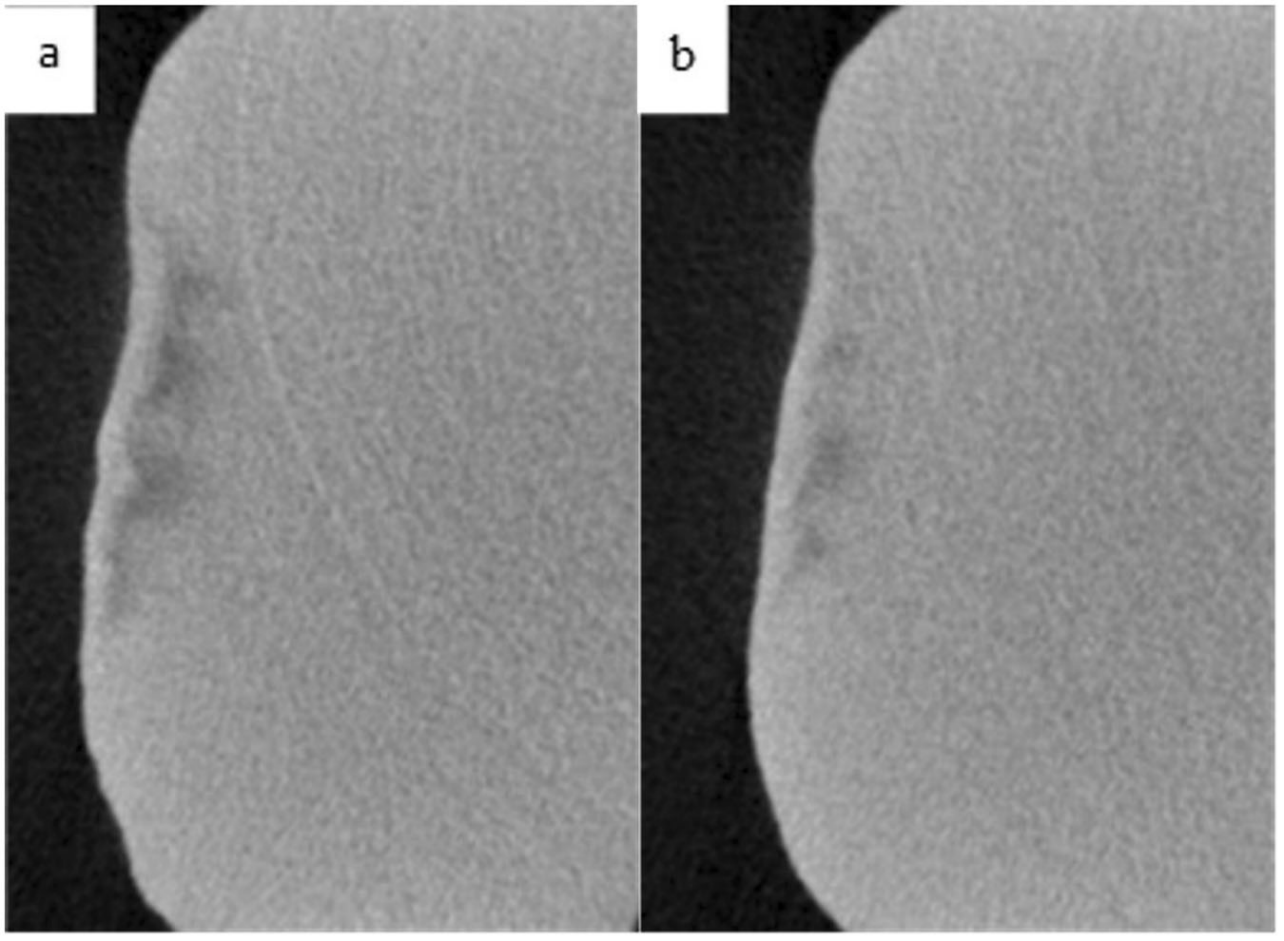
Remineralization of etched enamel MIH lesions with 20% hydroxyapatite toothpaste. Representative µ-CT images of etched enamel MIH lesions, before (**a**) and after (**b**) in-situ remineralization.

**Fig. 3:**
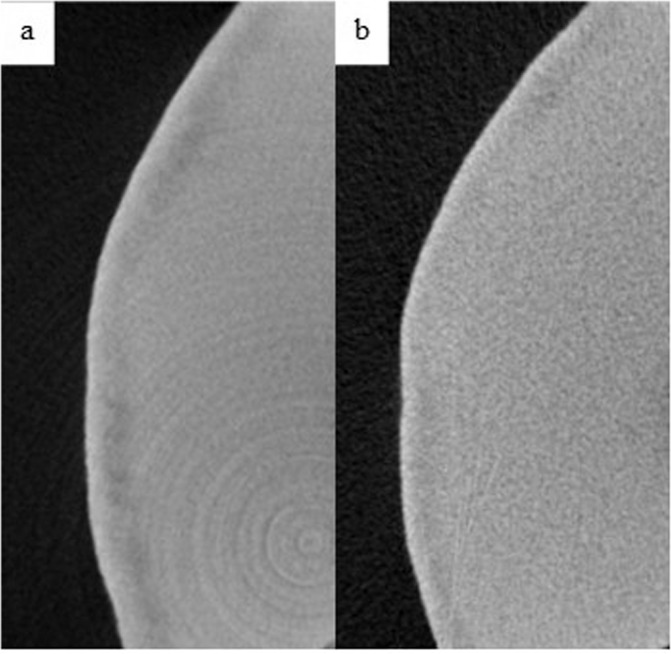
Remineralization of unetched enamel MIH lesions with 20% hydroxyapatite toothpaste. Representative µ-CT images of unetched enamel MIH lesions, before (**a**) and after (**b**) in-situ remineralization.

**Fig. 4:**
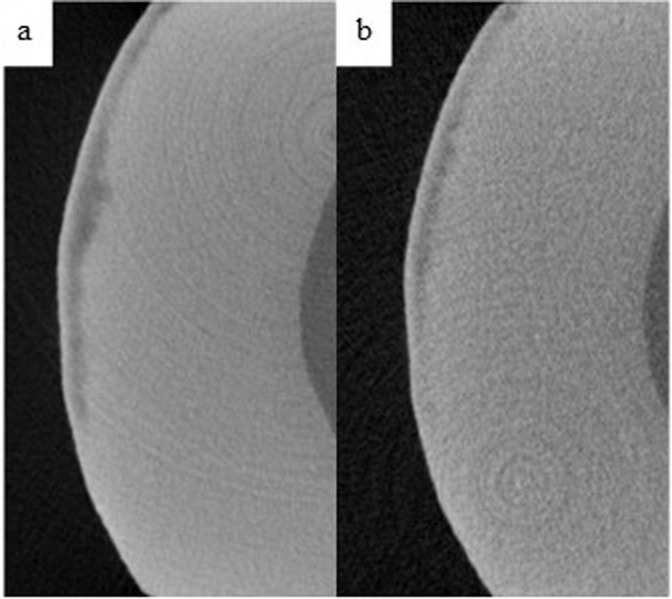
Remineralization of etched enamel MIH lesions with 1450 ppm NaF toothpaste. Representative µ-CT images of etched enamel MIH lesions, before (**a**) and after (**b**) in-situ remineralization.

**Fig. 5:**
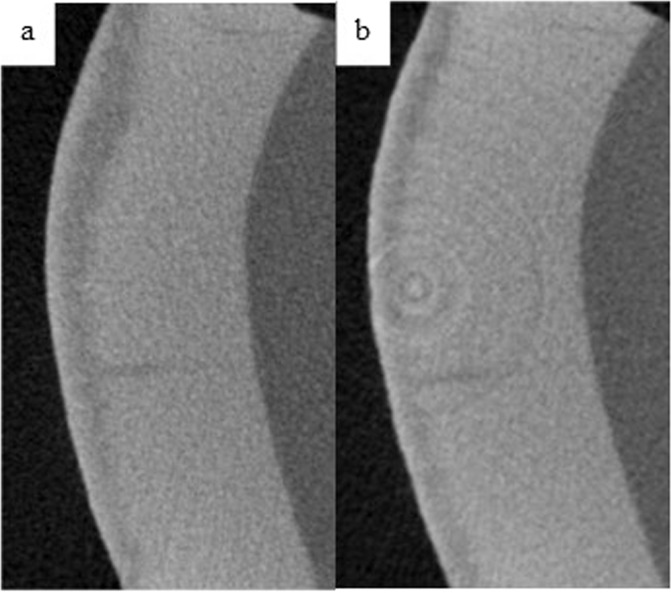
Remineralization of unetched enamel MIH lesions with 1450 ppm NaF toothpaste. Representative µ-CT images of unetched enamel MIH lesions, before (**a**) and after (**b**) in-situ remineralization.

Table 3 shows the intra-group comparisons for each of the product groups. In all groups, MD increased after the 14-days treatment with the respective toothpaste (Table 3). Figures 2 through 5 demonstrate the level of remineralization in each sample category (etched or unetched) in product group, showing clear evidence of remineralization of the MIH lesions. The result in Table 4 indicates that at two levels of comparisons (all data combined or etched data only), the difference in mean percentage remineralization between the two products were 11.38% (with combined data) and 12.43% (with etched data) with Cohen’s *d* large effect sizes (Table 4). With both figures being greater than the pre-established value for practical significance (≥10% with a Cohen’s *d* large effect size), a practically significant greater remineralization was achieved with HAP toothpaste when compared with fluoride toothpaste Figs. 3–5. However, with only the unetched data, HAP toothpaste achieved greater percentage remineralization than fluoride toothpaste, but the difference was only 6%, which is less than the pre-established 10%, thus there was no practical significant difference between the two products with regards to remineralization of unetched MIH lesions. Nevertheless, the Cohen’s *d* statistic of 0.777 shows a large effect size with both toothpaste formulations.

**Table 3:** Comparing pre-remineralization and post-remineralization mineral density (MD) for hydroxyapatite toothpaste (HAP) and fluoride toothpaste.

Outcome		Pre-treatment MD (g/cm^3^)		Post-treatment MD (g/cm^3^)		95% CI Diff.	*t*	*p*	*Cohen’s d*
		*M*	*SD*	*M*	*SD*				
Combined data	HAP	1.716	0.315	1.901	0.354	[0.053, 0.319]	2.874	0.004	0.564
	Fluoride	1.962	0.363	2.072	0.353	[0.017, 0.201]	2.458	0.011	0.482
Etched	HAP	1.700	0.345	1.943	0.339	[0.057, 0.428]	2.769	0.007	0.672
	Fluoride	1.916	0.396	2.026	0.410	[0.001, 0.242]	1.747	0.050	0.424
Unetched	HAP	1.744	0.265	1.822	0.390	[−0.107, 0.262]	0.971	0.180	0.324
	Fluoride	2.051	0.292	2.160	0.198	[0.002, 0.236]	1.978	0.042	0.659

*M* Mean, *SD* Standard Deviation, *CI* Confidence Interval, *t* t-test, *p* probability value, *Diff* Difference.

**Table 4:** Comparing the efficacy of hydroxyapatite (HAP) toothpaste and fluoride toothpaste on MIH lesion remineralization using percentage remineralization achieved with each product.

Outcome	HAP toothpaste		Fluoride toothpaste		95% CI Diff.	*t*	*Cohen’s d*
	*M*	*SD*	*M*	*SD*			
Combined data	26.02	20.68	14.64	9.60	[0.050, 3.35]	2.048	0.714
Etched	29.26	22.99	16.83	9.97	[−3.20, 28.06]	1.653	0.690
Unetched	16.62	5.74	10.62	8.13	[−5.21, 16.60]	1.204	0.777

*M* Mean, *SD* Standard Deviation, *CI* Confidence Interval, *t* t-test, *p* probability value, *Diff* Difference.

## Discussion

MIH constitutes a rapidly progressing defect which, in the absence of timely diagnosis and treatment, can negatively impact the patient’s quality of life [13]. Hypomineralized enamel is porous, immature and has a lower mineral content [2, 53]. These characteristics increase its susceptibility to many complications such as bacterial infiltration, rapidly evolving carious lesions, dental hypersensitivity, etc [1, 11, 12]. In fact, children with MIH are found to be ten times more likely to develop caries [12]. Thus, when MIH diagnosis is established, the implementation of preventive techniques is very important, to decrease the risk of caries and maximize the conservation of natural tooth tissue [3]. The mineral content of the enamel and its mechanical properties are directly correlated, and hypomineralized enamel has a lower mineral content and inferior mechanical properties [53]. Hence, an increase in the mineral density of the enamel can improve its properties, subsequently improving its resistance to breakdown and caries development. In particular, the use of remineralizing agents has been found to increase the mineral density of hypomineralized enamel in MIH affected teeth [17–20].

A commonly used remineralizing agent is fluoride due to the overwhelming evidences supporting its effectiveness in inhibiting tooth demineralization and promoting remineralization of initial caries [54]. However, both dental and systematic risks, such as fluorosis and toxicity, have been associated with increased fluoride exposure, particularly in children [27–29, 55]. Thus, the use of a remineralizing agent that is as effective as fluoride, but without health risks, may be a better alternative [47]. HAP particles are biomimetic and biocompatible, and they can be incorporated in oral hygiene products such as mouthrinses and toothpastes to provide several functions in preventive oral health care [44, 45, 47, 48]. In fact, studies have demonstrated the ability of HAP toothpastes to promote caries remineralization and prevent caries progression, with an effectiveness comparable to fluoride toothpastes [21–26, 47, 48]. However, to our knowledge, the present in-situ study was the first to evaluate the effectiveness of a HAP toothpaste in remineralizing MIH lesions in affected teeth and to compare it with a fluoride toothpaste.

The present study used an in situ-model, in which blocks derived from extracted MIH-affected teeth were mounted onto an intra-oral appliance that was worn by the participants. This model constitutes a bridge between laboratory and clinical settings, as it exposes the laboratory prepared samples to the oral environment and replicates the influence of therapeutic agents, saliva, and plaque, on the remineralization-demineralization process [56]. To induce biofilm retention on the surface of the MIH sample used in this study, polyester gauze was used to cover the surface of the blocks in contact with the oral environment. This model was used in previous studies as the gauze promotes the accumulation and conservation of plaque at the enamel surface, providing replica of areas at high caries risk and great acidic challenge [21, 23].

At baseline, the mean MD of the MIH-affected tooth blocks in the two product groups were 1.716 g/cm^3^ and 1.962 g/cm^3^, respectively. These values reflect the reduction in mineral density and mineral content of hypomineralized enamel in comparison to the MD of sound enamel that ranges between 2.4 g/cm^3^ and 2.8 g/cm^3^ across studies that measured it using x-ray microtomography [6, 51]. However, the MD of hypomineralized enamel in our samples fall within the range reported by Farah et al. who determined that the MD of mildly affected enamel is 2.24 g/cm^3^ and this number decreases even further to mean values between 1.67 g/cm^3^ and 1.93 g/cm^3^ in moderate to severe cases [5]. Accordingly, our measured MD values classify our sample in the moderate to severe category of MIH lesions. Both toothpaste formulations used in the present study achieved statistically significant increase in the MD of the MIH lesion after treatment (Table 3). This is in agreement with the report of a previous study that showed an increase in the mineral content of MIH affected teeth after treatment with CPP-ACFP toothpaste in vitro, and as such concluded that the mineral content of MIH defects can be improved after eruption [17].

In the present study, the samples assigned to each toothpaste group consist of etched and unetched samples. With either the HAP toothpaste or the fluoride toothpaste, remineralization was greater in the etched group than the unetched group (Table 4). This result is in agreement with studies that investigated the remineralization of other types of enamel hypomineralization [57, 58]. These studies found that pre-treating the hypomineralized enamel surface with an acid solution increased the rates of repair and remineralization and reduced lesion depth more significantly than without etching. Microscopically, enamel affected by MIH appears to have a thin, highly mineralized, less porous, surface layer covering the hypomineralized subsurface tissue [59]. Etching the lesion surface increases the porosity of the enamel surface, exposes more reactive crystals, increases the surface roughness of the enamel, and facilitates the diffusion of minerals to the deeper layers by removing deposits and creating microchannels to the body of the lesion [58]. These changes improve the rate of remineralization of both the surface layer and the underlying hypomineralized tissue [57]. Furthermore, MIH affected teeth have a higher protein content and proteins such as albumin are found in high concentrations within their organic phase [53, 60]. These proteins are implicated in the inhibition of crystal growth, and consequently the inhibition of pre-eruptive mineralization and post-eruptive remineralization [60]. To combat this barrier, a previous study used a 5% sodium hypochlorite (NaOCl) solution as a deproteinizing agent to pre-treat the MIH sample before exposure to remineralizing agent in vitro [17]. The experimental group pre-treated with NaOCl had the greatest absolute gain in minerals. However, no other study demonstrated the effectiveness of this procedure in vivo.

The results of the present in situ study showed that each of the two toothpaste formulations achieved a mean percentage remineralization that is greater than zero, thus rejecting the first null hypothesis. This is in line with previous studies that demonstrated the effectiveness of fluoride in remineralizing different types of hypomineralized enamel [3, 14, 17–20, 54, 61, 62]. In particular, some authors have investigated the benefit of a regular application of fluoride on the remineralization of MIH lesions [18, 20, 63]. Restrepo et al. [63], for example, reported no signifiant increase in the MD of MIH lesions, measured by quantitative laser fluorescence (QLF), after the application of fluoride varnish. However, other studies showed a significant remineralization effect of fluoride application on MIH lesions, which is comparable to the results of the present study [18, 20]. On the other hand, HAP toothpastes were shown in different in vitro and clinical studies to promote the remineralization of caries lesions via two mechanisms [21, 22, 26, 47, 48]. Firstly, HAP particles can dissolve in bacterial biofilms and increase the bioavailable calcium and phosphate ions in the immediate environment of the tooth surface, and secondly, the HAP particles can be directly deposited in the microporosities of demineralized tissue, where they promote crystal deposition and growth by continuously attracting calcium and phosphate ions from the surrounding remineralization solution [42–44]. It is believed that these mechanisms may have applied in the present study. Nevertheless, the findings of the present study extend the scope of application of HAP toothpaste to the therapeutic management of MIH lesions as well.

The present study demonstrated that the HAP toothpaste induced practically significantly greater remineralization of MIH lesions than fluoride toothpaste, thus rejecting our second null hypothesis. With both the combined data and only the etched data (but not with the unetched data), the difference in the percentage remineralization achieved by the two products exceeded the pre-set threshold of 10% for practical significant difference. We used the concept of practical significance here because research on quantitative analysis have reported on the limitations of statistical significance and p-values and why they should not be used alone in comparative studies even when there is statistical significance difference, rather they should be supplemented with practical significance to determine if this difference is relevant in the real world [49, 50]. The studies demonstrated that statistical significance is influenced by sample size, while practical significance is not, because it uses effect sizes instead. They further proved that statistical significance focuses on showing the existence or absence of an effect, with an interpretation limited to statistical relevance, whereas practical significance determines the magnitude of the effect, to establish if its meaningful in the real world. Thus, in the present study, using the concept of practical significance offers a better perspective on how impactful the difference between the two toothpastes is in the clinical practice, and shows the superiority of the HAP toothpaste in remineralizing MIH. However, it is pertinent to mention that previous studies comparing the efficacy of HAP and fluoride toothpastes in remineralizing caries lesions showed a comparable efficacy between both products but no statistically significant difference was observed [25, 26]. Furthermore, in vivo and in situ clinical trials have long demonstrated the non-inferiority of toothpastes based on HAP to different concentrations of fluoride toothpastes [21, 23, 33, 48, 64].

The absence of any adverse effects either reported by the subjects or observed clinically in the present study is in agreement with previous clinical studies that reported the absence of side effects following the use of different concentrations of HAP in different oral hygiene products [21, 23, 33]. Therefore, it can be concluded that a potential increase in the dose of HAP in toothpastes can be considered safely, with an aim to exponentiate effectiveness of these toothpastes.

## Conclusions

- This in situ study demonstrated that the tested fluoride-free hydroxyapatite toothpaste can remineralize MIH lesions.
- The HAP toothpaste achieved practically significantly higher remineralization than the fluoride toothpaste.
- Pre-treating the tooth surface with acid-etchant enhanced remineralization of MIH lesions.
- In the future, in vivo randomized clinical trials are needed to further establish the scope of application of these findings in children with MIH. It is also important to investigate procedures, such as different surface pre-treatment techniques, and their potential in optimizing the remineralization rate to achieve better outcomes.
